# Virtual Reality Customized 360-Degree Experiences for Stress Relief

**DOI:** 10.3390/s21062219

**Published:** 2021-03-22

**Authors:** Miguel A. Vaquero-Blasco, Eduardo Perez-Valero, Christian Morillas, Miguel A. Lopez-Gordo

**Affiliations:** 1 Department of Signal Theory, Telematics and Communications, University of Granada, Calle Periodista Daniel Saucedo Aranda, s/n, 18014 Granada, Spain; miguelvaquero@ugr.es; 2 Research Centre for Information and Communications Technologies (CITIC), University of Granada, Calle Periodista Rafael Gómez Montero, 2, 18014 Granada, Spain; edu@ugr.es (E.P.-V.); cmg@ugr.es (C.M.); 3 Department of Computer Architecture and Technology, University of Granada, Calle Periodista Daniel Saucedo Aranda, s/n, 18014 Granada, Spain

**Keywords:** virtual reality, EEG, emotions, stress

## Abstract

The latest studies in virtual reality (VR) have evidenced the potential of this technology to reproduce environments from multiple domains in an immersive way. For instance, in stress relief research, VR has been presented as a portable and inexpensive alternative to chromotherapy rooms, which require an adapted space and are expensive. In this work, we propose a portable and versatile alternative to the traditional chromotherapy color-loop treatment through four different 360-degree virtual experiences. A group of 23 healthy participants (mean age 22.65 ± 5.48) were conducted through a single-session experience divided into four phases while their electroencephalography (EEG) was recorded. First, they were stressed via the Montreal imaging stress task (MIST), and then relaxed using our VR proposal. We applied the Wilcoxon test to evaluate the relaxation effect in terms of the EEG relative gamma and self-perceived stress surveys. The results that we obtained validate the effectiveness of our 360-degree proposal to significantly reduce stress (*p*-value = 0.0001). Furthermore, the participants deemed our proposal comfortable and immersive (score above 3.5 out of 5). These results suggest that 360-degree VR experiences can mitigate stress, reduce costs, and bring stress relief assistance closer to the general public, like in workplaces or homes.

## 1. Introduction

Modern advancements in Virtual Reality (VR) systems have enabled the use of this technology in many different contexts. VR has become a useful assisting tool in multiple environments, such as disorder therapies [1,2,3], rehabilitation processes [4,5,6,7], marketing [8,9,10], industry [11,12,13], or safety and industrial trainings [14,15,16]. Nonetheless, there are still many areas like relaxation therapies, where traditional approaches are preferred. Chromotherapy is a color-light-based therapy widely used to reduce psychological stress. This therapy is conducted in a comfortable room, where light and sounds are adjusted to induce a relaxation state [17]. However, chromotherapy still remains undiscovered for a broad part of the population. In most cases, people prefer to relax through experiences they are attracted to, such as going to the beach or simply being alone in a quiet place. Furthermore, chromotherapy requires investments in terms of physical space, deployment costs, and maintenance.

In this work, our aim was to examine the feasibility of VR-based customized 360-degree experiences for stress relief as an alternative to chromotherapy. Participants were stressed and then conducted through a relaxation phase where they selected one of the four 360-degree experiences available. We recorded the electroencephalography (EEG) activity of the participants throughout the experiment, and asked them about their self-perceived stress level (SPSL) via surveys. We used a commercial VR device, the Oculus Quest head-mounted display (HMD), to implement four different 360-degree virtual experiences: a lone beach, a cave with a water cascade, a space trip, and an aurora borealis sight. In each scenario, we included relevant background sounds, either to emulate a characteristic environmental sound (like in the beach and cascade experiences) or to render a relaxing ambient (like in the space and aurora experiences). Since we aimed to evaluate the effectiveness of the experiences preferred by the participants, they were asked to select one of the four VR scenarios during the relaxation stage of the study. Previous studies have assessed the potential of VR to help deal with stress [18,19,20]. For instance, in [18], we demonstrated that a VR application that emulates color-light therapy can achieve the same performance in terms of stress relief as chromotherapy. In [21], the authors used a cave automatic virtual environment (CAVE) system to create a virtual natural environment, including a water stream and nature sounds to enhance relaxation.

Multiple biomarkers have been widely validated in literature for stress assessment. The most widespread are electrocardiography (ECG) [22,23,24], electroencephalography (EEG) [25,26,27], galvanic skin response (GSR) [28,29,30], and cortisol, which is widely accepted as a reference for stress evaluation [31,32]. Among these, EEG is a suitable alternative if high temporal resolution is required [33]. This technique has been used in recent studies to detect stress in construction workers [34], perform a three-level classification of stress [35], or for stress level assessment in response to music tracks [25]. To evaluate our proposal, we used the relative gamma (RG), a power spectral density (PSD)-based EEG stress marker. According to previous works, there is a relationship between RG and the stress level felt [17,18,36,37]. It is worth mentioning that this relationship can be either directly proportional [17], inversely proportional [36,37], or show both trends [18,33]. This effect has to be considered during signal processing, but the reasons behind it still remain undetermined. Other EEG biomarkers used for stress assessment include spectral power [38] and asymmetry in alpha [39,40] and other EEG bands [41].

To sum up, using 360-degree VR experiences, in this study we provide an attractive and portable alternative to bring stress relief assistance closer to a broad audience. This solution could be implemented in contexts such as office breaks, educational time-out periods, hospitals, travels, or at home. Lastly, this kind of approach has not been widely explored in literature yet, nonetheless, some studies have investigated the potential of VR for stress relief [42,43,44]. Our study evidences the relaxation capabilities of VR technologies by providing the participants with a set of personalized 360-degree virtual experiences that represent a portable and inexpensive approach to stress relief.

## 2. Methodology

### 2.1. Participants

Twenty-three healthy participants (mean age 22.65 ± 5.48, age range from 18 to 40; 8 males, 14 females, and 1 non-binary) were recruited two weeks before the beginning of the study. They signed an informed consent and they were asked not to take any relaxant or stimulant the day before the study. All the participants were students from the University of Granada, they participated voluntarily in this study, and did not receive any reward. Only participants without mental disorders or health issues were considered. Each participant was conducted through a single session that lasted approximately 30 min. During the session, participants had the chance to end their involvement in the study at any time in case they felt uncomfortable. The full data capture was completed in three weeks.

### 2.2. Experimental Procedure

**Figure 1:**
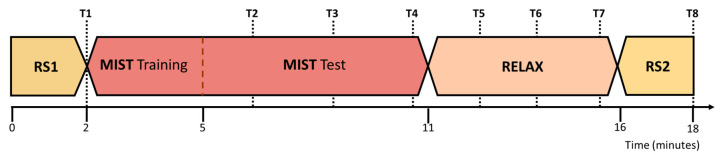
Time flow of the experimental session. First, the participants completed a two-minute eyes-closed resting state period (RS1). This process was repeated at the last stage of the experimental procedure (RS2). Then, the participants were stressed through a test designed to induce psychological stress by means of arithmetical operations (MIST). Finally, they relaxed through a 360-degree virtual experience (RELAX) for 5 min. The experimental procedure lasted about 18 min. During the session, we performed multiple surveys to gather the self-perceived level of stress of the participants (T1–T8). MIST: Montreal imaging stress task.

Before the onset of the experiment, we equipped the participants with an EEG acquisition system for the recording of the electrophysiological activity of the brain. EEG systems have been widely used in fields like communications [45,46], attention detection [47], and entertainment [48]. Once the participants were equipped with the EEG acquisition system, they were briefed about the tasks they had to complete during the session. The different stages of the session are illustrated in Figure 1.

**Figure 2:**
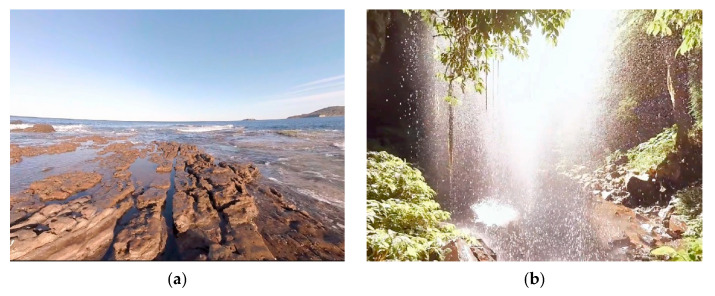

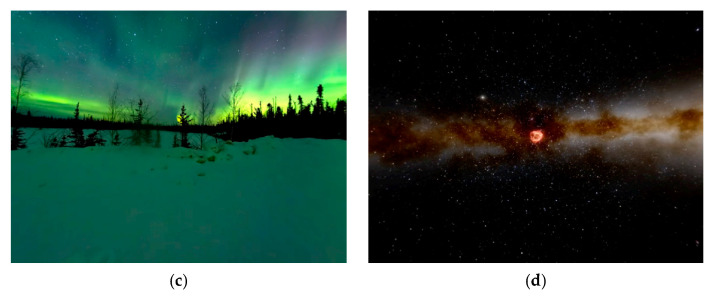
In-game view screenshots of the different 360-degree scenarios. (**a**) Beach. (**b**) Cascade cave. (**c**) Aurora Borealis. (**d**) Space.

First, the participants performed a two-minute eyes-closed resting state period (RS1). Then, we stressed them using an adaptation of the Montreal imaging stress task (MIST) [49], an arithmetical test designed to induce psychological stress that has been validated by multiple studies [50,51,52]. Subsequently, the participants selected one of the four 360-degree experiences available and went through it using a VR HMD (RELAX). This stage was carried out in a room with low ambient light and controlled temperature. Participants remained seated in a comfortable chair placed in the center of the room while they were equipped with the HMD. They were instructed not to move in order to minimize EEG artifacts, and they selected their preferred relaxation experience from a menu list using the HMD controller. This list included four scenarios (see Figure 2). Lastly, the participants completed a final two-minute eyes-closed resting state period (RS2). For the resting state periods and the relaxation phase, the participants remained alone in the room and they were monitored from the outside. During the session, we conducted multiple surveys to gather the self-perceived stress level of the participants (T1-T8), and we recorded their EEG activity to obtain biomarkers of their stress level. Once the session concluded, the participants completed a survey to evaluate the VR experience in general terms and with respect to comfort and immersion. We also asked the participants if they would repeat the experience.

To avoid interruptions during the experimental procedure, we incorporated surveys T2–T4 into the MIST software application, and we integrated surveys T5-T7 into the 360-degree VR experience and instructed the participants to answer using the HMD controller.

### 2.3. Experimental Setup

We developed the virtual experiences for this experiment using Unity software (2020 version). We implemented each scenario using a 360-degree video as background of the Unity scene. Then, we placed the VR in-game view in the center of the scene and configured it to allow the movements and rotations of the Oculus Quest HMD. Additionally, we programmed the SPSL surveys to appear periodically in the in-game view so the participants were able to answer using the HMD controller. At the beginning of the relaxation stage, all the virtual experiences available were displayed in a menu to facilitate its selection. We included four immersive experiences: a solitary beach, a cave with a cascade, a space trip, and the sight of an aurora borealis. Screenshots of all the experiences are shown in Figure 2.

To record the EEG signals, we used the Versatile semi-dry EEG system (Bitbrain) [53] at a sampling rate of 256 Hz. We placed eight electrodes at Fp1, Fp2, F5, F6, Fz, Cz, O1, and O2 positions of the 10–20 International System. However, we considered only Fp1, Fp2, F5, and F6 for this study. We selected these locations in accordance with previous successful studies on emotions assessment using EEG [18,54,55,56]. The electrodes were grounded using an extra electrode placed at equal distance between Fpz and Fz, and referenced to the left ear lobe.

**Figure 3:**
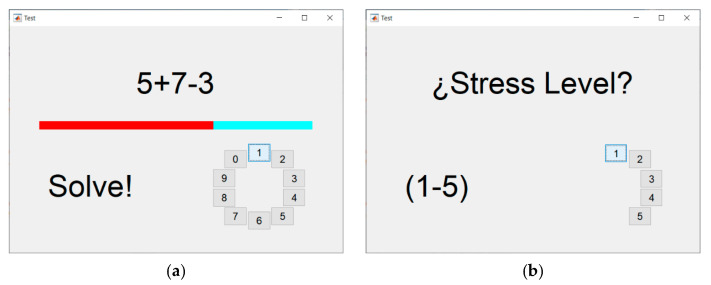
Montreal imaging stress task (MIST) interface during the test phase. (**a**) View displayed for each arithmetical operation. (**b**) View displayed during the stress surveys.

With respect to the MIST, we implemented this test as a graphical interface using MATLAB R2016a (MathWorks). The test consisted of a series of arithmetical operations based on combinations of additions, subtractions, multiplications, and divisions. First, the participants completed a three-minute training phase with no time limit to solve each operation. Then, they performed a six-minute test phase, with a time limit to complete each operation. The MIST interface displayed the time limit as a progress bar over each operation. Moreover, the interface also showed the accumulated success rate of the participant as a second progress bar. The success rate was intentionally displayed to induce pressure on the participant. Additionally, the technician in charge of the experiment entered the room to verbally put pressure on the participants on three occasions during the test phase. Likewise, we integrated three SPSL surveys into the MIST interface so the participants could report their self-perceived level of stress during the test phase (see Figure 3). To complete the MIST, we instructed the participants to remain seated and to use the touchscreen of the laptop with their dominant hand to solve the operations. The MIST had a duration of 9 min, including the train and the test phase.

Finally, to assess the self-perceived stress level of the participants, we adapted the perceived stress scale (PSS) [57] to minimize the interaction with the participants and the time required to answer. Each survey consisted of one single question: “what is your stress level from 1 to 5? being 1 the minimum level and 5 the maximum level”. The surveys were performed at diverse points of interest: T1, after the first resting state (RS1); T2 and T3, 120 and 240 s after the start of the MIST test phase, respectively; T4, at the end of the MIST; T5 and T6, 90 and 180 s after the onset of the relaxation phase, respectively; T7, at the end of the relaxation phase; T8, after the second resting state (RS2). We chose this separation between the surveys as a trade-off between stress tracking and participant disturbance during the experimental procedure.

### 2.4. Signal Processing

First, we concatenated the EEG recordings of all the experiment stages to process them jointly. We excluded the MIST training stage from this concatenation. With regard to the resting state periods, we only considered the central minute. We applied a third-order zero-phase shift bandpass Butterworth filter (bandpass 1–50 Hz) to the EEG signals. Then, we used a notch filter (stopband 48–52 Hz) to remove electric couplings [17,18]. Subsequently, we divided the EEG signals into two-second epochs without overlapping to perform a spectral analysis. For each epoch, we considered as outliers the channels with an amplitude absolute value above 75 µV and zeroed them. We selected this threshold based on meticulous visual inspection of the EEG according to previous studies [58,59,60]. Then, we detrended and z-scored each epoch, and we estimated the power spectral density (PSD) in several frequency bands (see Table 1). Subsequently, for each band, we averaged the PSD from all the channels.

Based on the PSD, we obtained the relative gamma (RG). The RG is a biomarker linked to stress that is obtained as the power ratio between the power in the Gamma band and the power in the slow rhythms (Alpha and Theta) (see Equation (1)). We selected the RG since the relationship between this biomarker and the stress level has been evidenced in previous studies in the literature [17,18,36].

RG = P_Gamma/_(P_Alpha_ + P_Theta_) (1)

To equal the length of the RG signals for all the participants, we resampled them to 390 samples and then smoothed them using a moving average filter with a 15-sample span. This 390-sample length corresponds to the 13-min part of the 18-min EEG recording that we considered for the analysis (see Equation (2)). Therefore, the filter window included 15 points, the center sample, the 7 previous samples, and the 7 subsequent samples. Considering that each sample corresponds to a two-second epoch, the filter will shift the fluctuations in RG around 15 s with respect to the expected instant (7 samples × 2 s per sample).

390 _epochs_ = (13 _min_ · 60 _s/min_)/2 _s/epoch_ (2)

Finally, through visual inspection, we detected the participants whose RG showed an inverse relationship with the stress level felt. As indicated in a previous study [18], the RG signals from these participants were inverted in order to be averaged with the data from the rest of the participants, whose RG showed a direct relationship with the stress level.

### 2.5. Statistical Analysis

To perform the statistical analysis, we estimated the grand average of the RG across all the participants, and we also calculated the standard error of the mean (SEM). Furthermore, we computed the mean of the SPSL reported by the participants via the SPSL surveys. Then, we used the Wilcoxon signed-rank test to determine if there were significant differences among the mean stress perceived by the participants along the different phases of the experimental procedure. We applied this test as the data did not pass the Lilliefors normality test. We also used the Wilcoxon rank sum test to check if there were significant differences between the four 360-degree experiences in terms of the SPSL reported by the participants during the relaxation phase. For all the statistical tests, we considered a significance level of 0.05 (*α* = 0.05). Moreover, we obtained the cross-correlation coefficient for the SPSL survey answers and the mean value of the RG during the minute that preceded each survey, to evaluate the relationship between these two stress measures.

Finally, for the surveys regarding the user experience with the 360-degree virtual application, we calculated the mean value and standard deviation of the answers provided by the participants. This survey assessed three aspects about the 360-degree relaxation experience the participants were conducted through, namely, the immersion level, the comfort level, and a general evaluation of the experience.

## 3. Results

It is worth mentioning that, due to noise issues in the EEG recordings, we discarded the data corresponding to four participants (S07, S08, S11, and S14). With respect to artifact removal, some epochs were rejected for each participant. A maximum of 12% of the epochs were removed for a single participant (S17), although in most cases, less than 5% of the epochs were rejected. As outlined in the previous section, the RG biomarker can show either an inversely or directly proportional response to the stress level felt. In this study, we found that the RG of four participants was directly proportional to the stress level (S06, S10, S20, and S22) and the RG of the rest of the participants showed an inverse response.

**Figure 4:**
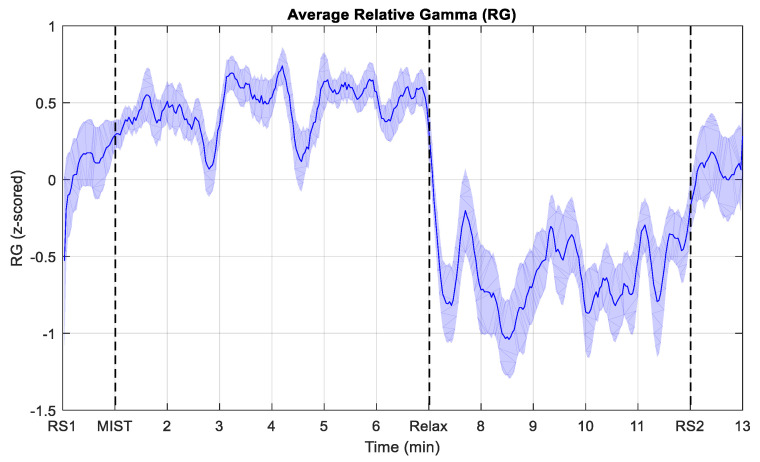
Evolution of the average relative gamma (RG) during the experiment. To obtain the average, the RG from the participants whose response was inversely proportional to the stress level felt was inverted before averaging. RS1 and RS2 indicate the start of the resting state periods. MIST corresponds to the beginning of the stress session (only the six-minute test phase). Relax refers to the start of the relaxation phase. The shaded area corresponds to the standard error of the mean (SEM).

Figure 4 depicts the RG averaged across all the participants. To obtain this curve, we first inverted the RG of the participants whose response was inversely proportional to the stress level and then estimated the average.

**Figure 5:**
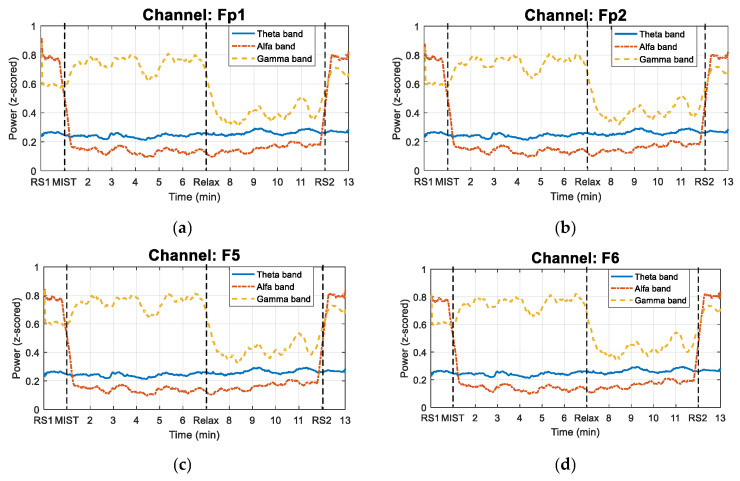
Power changes in Theta, Alpha and Gamma bands for each of the four electrodes considered in the study for subject S02. The solid line represents the power evolution in the Theta band. The dash-dotted line indicates the time evolution of the power in Alpha band. The dashed line corresponds to the power changes in Gamma band. (**a**) Fp1 channel. (**b**) Fp2 channel. (**c**) F5 channel. (**d**) F6 channel.

Figure 5 displays the time evolution of the power in the different bands (Theta, Alpha and Gamma) used to obtain the RG. This figure shows the power changes of the signals obtained in each of the four electrodes that we used in the study. All the curves correspond to subject S02.

**Figure 6:**
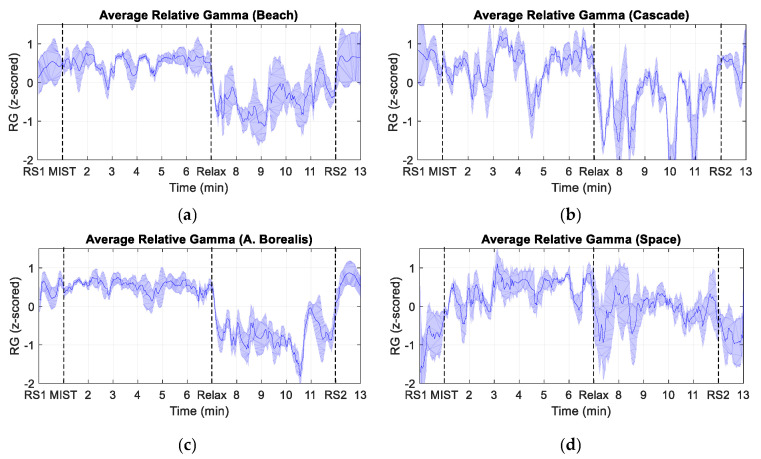
Time evolution of the average relative gamma for the four different 360-degree experiences. The shaded area corresponds to the standard error of the mean (SEM). (**a**) Beach experience. (**b**) Cascade cave experience. (**c**) Aurora Borealis experience. (**d**) Space experience.

Figure 6 illustrates the average RG of the participants for each of the four different 360-degree experiences. The proportion of the total number of participants that selected each experience was: ~35% for Aurora borealis, ~30% for Space, ~22% for Beach, and ~13% for Cascade cave.

**Figure 7:**
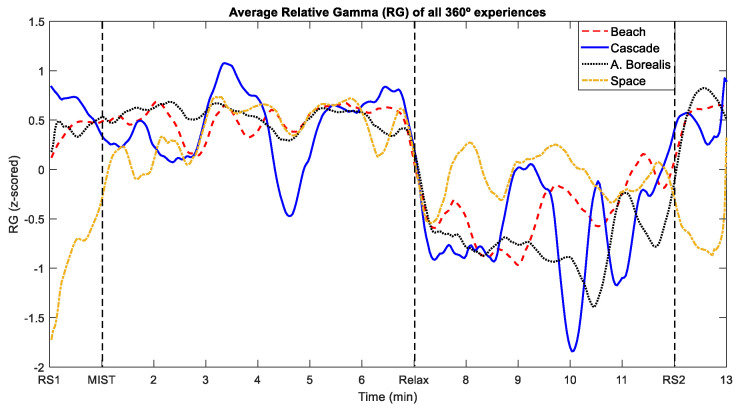
Comparison of the time evolution of the average RG for the four different 360-degree experiences. The dashed line corresponds to the Beach experience. The solid line represents the Cascade cave experience. The dotted line corresponds to the Aurora Borealis experience. The dash-dotted line represents the Space experience.

Figure 7 shows the RG averaged across participants for each of the four different 360-degree experiences. This figure displays all the graphs presented in Figure 5, but we removed the SEM shaded area and smoothed the curves to facilitate their view.

Table 2 presents the results yielded by the Wilcoxon rank sum tests performed to assess the differences among the SPSL survey answers during the relaxation phase, for the different 360-degree experiences. No significant differences were found in any of the tests performed.

Table 3 shows the *p*-values obtained with the Wilcoxon signed-rank tests that we applied to compare the SPSL survey answers during the different phases of the experimental procedure.

**Figure 8:**
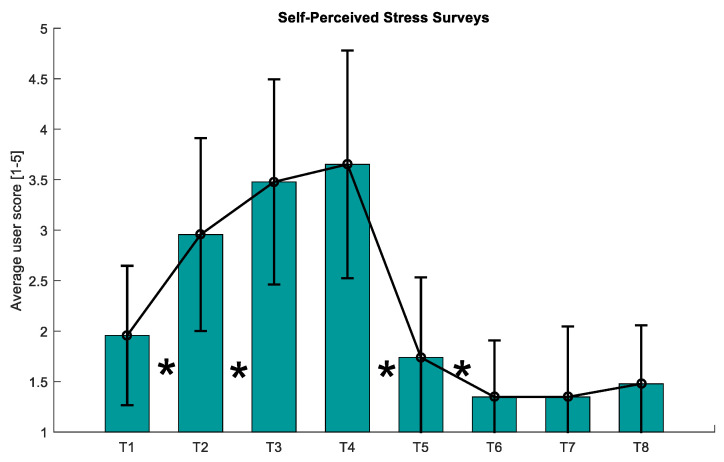
Average of the SPSL answers given by the participants. Vertical black lines indicate the standard deviation of the mean. The asterisks (*) indicate statistically significant differences (*p*-value < 0.05) between T1–T2, T2–T3, T4–T5, and T5-T6. SPSL: self-perceived stress level.

Figure 8 displays the average across all the participants of the SPSL survey answers throughout the experiment.

**Figure 9:**
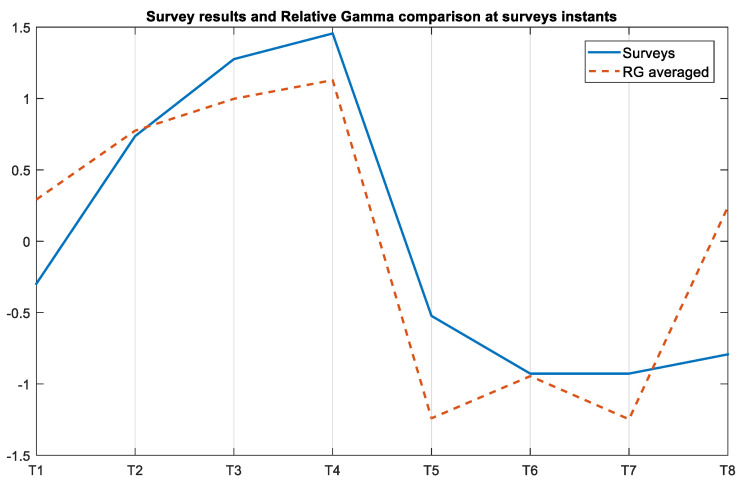
Normalized average of the SPSL answers and the average RG across participants at the eight survey instants. We computed the RG values as the mean of the RG curve in the minute that preceded each survey.

Figure 9 jointly shows the normalized average across participants of the SPSL survey answers and the RG at the instants those surveys were conducted (T1–T8). We estimated the RG values as the mean value of the RG in the minute that preceded each survey. We also calculated the Pearson’s correlation coefficient (*PCC*) between the SPSL answers and the average RG at T1-T8, and obtained a value of 0.8417 (*CI* = [0.3367, 0.9707]).

**Figure 10:**
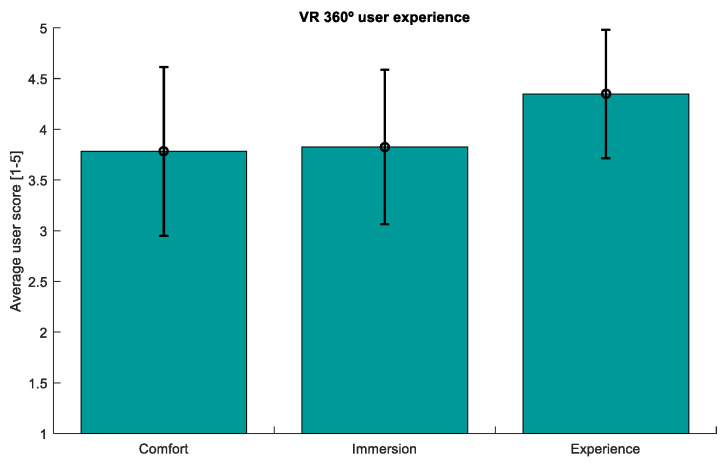
Average across participants of the user experience survey answers. This survey evaluated three aspects of the 360-degree experience: comfort, level of immersion, and general experience. The Y axis represents the mean score of the three aspects, from 1 to 5. Vertical black lines indicate the standard deviation of the mean.

Finally, Figure 10 represents the average of the answers to the 360-degree user experience surveys across all the participants. With regard to the question that we asked to discern whether participants would repeat the experience, all the participants claimed they would repeat it.

## 4. Discussion

The aim of this work was to assess the feasibility of 360-degree virtual reality experiences for stress relief as an alternative to chromotherapy. In this paper, we obtained the RG to evaluate the stress level of the participants during a stress–relax session. We designed our approach based on EEG, that provides high temporal resolution, and short SPSL surveys to gather the perception of the participants and minimize the time required to answer. The results that we obtained evidence that our 360-degree experiences reduced stress in a comfortable and immersive way.

In relation to the evolution of the RG, as expected, it can be noticed that the curve starts and ends near the zero value (see Figure 4), which suggests that the resting state periods induced a baseline stress level in the participants. The curve also shows a positive trend during the MIST phase, and a negative tendency during the relaxation period. As expected, the most intense drop of the RG takes place just after the beginning of the relaxation phase (circa minute 7). Furthermore, the curve presents three local minima of the RG in the MIST phase (circa minute 3, 5, and 6), and three local maxima in the relaxation phase (circa minute 8, 9, and 11). This behavior may be explained by the SPSL surveys conducted throughout these two phases of the study. This may suggest that the surveys had a strong effect on the stress level of the participants. In addition, it should be remarked that the fluctuations of the RG curve associated to the SPSL surveys are not exactly aligned with the instant these surveys were performed. This is an expected effect of the moving average filter that we applied to smooth the signals. The effect of the smoothing filter can also be noticed at the beginning of the different phases of the experimental procedure. In addition, it is worth noting that multiple studies have suggested that the Alpha band may be affected by the performance of mathematical operations [61,62,63], which would question the reliability of the RG biomarker. Nonetheless, when we performed signal processing, we observed that the fluctuations in Alpha during the MIST stage were negligible (see Figure 5).

With regard to the different virtual experiences that we implemented in this work, shown in Figure 6 and Figure 7, the intense drop of the RG just after the start of the relaxation phase is notable for all of them. Furthermore, as indicated in Table 2, no significant differences were found among the four virtual scenarios for any of the surveys conducted throughout the relaxation phase. These results indicate that the different customized 360-degree experiences reduced the stress level with similar performance. It should be noted that the RG curve associated to some of the experiences presents more fluctuations than the others, but this could be explained by the number of participants that chose each experience.

Visual inspection of the SPSL survey answers (see Figure 8) evidences that the stress level reported by the participants follows the same trend as the RG curve. Likewise, we found statistically significant differences between the answers of T1-T2, T2-T3, T4-T5, and T5-T6, as reported in Table 3. This proves that the stress level of the participants increased during the MIST and decreased during the 360-degree virtual experiences. Furthermore, according to Figure 9, there exists a high correlation between the RG and the SPSL. Indeed, the correlation coefficient between the two variables (PCC = 0.8417) supports the use of RG as a trustful stress biomarker. Also, according to the user experience survey answers, our proposal was considered comfortable and immersive by the participants (see Figure 10).

In relation to other state-of-the-art works, the results that we obtained are in line with recent studies that assessed the potential of VR for stress relief. For instance, in [42,43], the authors demonstrated the relaxing potential of vegetation in an urban virtual environment and the relaxing effects of watching a virtual forest video on stress relief, respectively. In the same way as our methodology, the authors of these studies designed a protocol to stress the participants through arithmetic operations. They also measured physiological stress from markers such as the skin conductance levels (SCLs) or the heart rate variability (HRV). Compared to our EEG-based approach, skin conductance and heart rate present lower temporal resolution. This advantage of EEG is crucial in the potential development of real-time stress monitoring systems. Furthermore, in [43], the authors placed electrodes on the fingers of the participants, and an electrodermal activity transmitter on their wrist. This type of montage is more prone to artifacts due to movements of the higher limbs compared to our semi-dry four-electrode setup. In addition, the authors assessed the psychological state of the participants through validated scales like the Positive and Negative Affect Schedule (PANAS) [64] to evaluate their mood. Although this scale provides insights about the psychological state of the participants, it is composed of 20 questions. Thus, the length of this evaluation may interfere with the relaxation state of the participants. For this purpose, we used a shorter psychological assessment based on PSS.

Moreover, some of the features that are relevant for VR stress assessment have been overlooked in other studies. These features include physiological measurements to support the results, robust scales to evaluate the mood of the participants, or standardized protocols to equally stress the participants. For instance, the authors of [65,66,67,68] developed a virtual environment by combining VR, audio and olfactory stimuli, or breathing techniques to promote relaxation; however, they did not report an established methodology to equally stress the participants. A protocol like the MIST, that we implemented in this study, is important since it allows the investigators to attribute the differences in stress levels between stress phase and relaxation phase to the VR environments that they implemented. In [69], the authors used an HMD to induce relaxation through a 360-degree video of a local city beach, while at the same time they measured the electrodermal activity of the participants. Nevertheless, this biomarker did not provide meaningful insights in terms of stress, conversely to our study, where the relative gamma supported the SPSL progression. Furthermore, they used a foot tub to offer more realism during the experience, which may diminish portable capabilities compared to our approach, that only included an HMD to stimulate the participants. In [70], the authors followed a methodology that included a stress phase and a subsequent VR experience. Nevertheless, no physiological parameters were monitored, which led the authors to the conclusion that EEG should have been acquired for further assessment of their VR proposal.

Lastly, it is not feasible to perform a quantitative comparison between the results yielded in this study and those that we obtained in a previous VR-based chromotherapy work [18], as the experimental conditions differ. However, a qualitative comparison of the RG trends and SPSL surveys derived in both studies suggests that the two approaches helped relief stress similarly. This implies that traditional color-based relaxation treatments may be replaced by customized VR experiences, which might give these therapies a greater public appeal. Regarding the participants, the dataset that we gathered held data from 23 participants. A higher number of participants might have reduced the confidence intervals of the correlation coefficients, although our results are good enough to support our conclusions. Moreover, the results that we obtained suggest that the duration of relaxation therapies could be reduced, since only a few minutes are enough to reduce the stress level (see the drop produced in the RG curve after the onset of the relaxation phase in Figure 4). This finding could benefit environments such as workplaces or schools, as short time-outs could be utilized to relief stress, which would likely increase productivity and focus.

## 5. Conclusions

In this paper, we have demonstrated the feasibility of VR-based 360-degree experiences for stress relief. Our main purpose was to reduce the costs associated with traditional chromotherapy rooms, and to offer a new and attractive alternative to relieve stress based on individualized experiences to avoid boredom or rejection to traditional relaxation methods. For this purpose, we followed a robust methodology to elicit stress in 23 participants, and then relaxed them with our 360-degree proposal. We gathered the self-perceived stress level of the participants via surveys conducted at eight key moments of the experimental procedure. Furthermore, we recorded their EEG activity to obtain a stress biomarker, the relative gamma. According to our results, our proposal only needs a few minutes to reduce the stress level, and the experience was described as comfortable and immersive by the participants, who also claimed that they would repeat it. This proposal presents several advantages compared to other relaxation methods. First, traditional relaxation therapies, like chromotherapy rooms, are expensive in terms of space and initial investments. These costs far exceed those associated with a commercial head-mounted display. Also, our proposal can be exported to multiple commercial devices, thus it offers a level of ubiquity and availability that cannot be achieved with conventional solutions. In summary, the results we obtained validate our 360-degree proposal as a new and reliable stress relief alternative. Its attractiveness and low cost make our proposal suitable for several environments, such as workplaces, schools, therapy clinics, and hospitals. For instance, a school time-out could be used by students to relax using a commercial HMD and their favorite 360-degree experience. Finally, for future studies, we propose the use of the RG to model the stress level through regression, based on the high correlation shown between the RG and the SPSL survey answers (*PCC* = 0.8417). This could allow the prediction of the stress level in real-time applications. Also, further studies should consider a combination of the RG with other stress biomarkers (GSR, ECG, cortisol, etc.) to gain new insights about this biomarker.

## Figures and Tables

**Table 1:** Frequency bands used to estimate the PSD.

Freq. Band	Delta	Theta	Alpha	Beta	Gamma
Range (Hz)	1–4	4–8	8–13	13–25	25–45

**Table 2:** *p*-values of the Wilcoxon rank sum tests performed to compare the SPSL survey answers during the relaxation phase for the different 360-degree experiences. The rows indicate the experiences compared and the columns display the SPSL survey instant.

	T5	T6	T7
Beach–Cascade	1.0000	1.0000	1.0000
Beach–Borealis	0.4988	0.2378	0.7832
Beach–Space	1.0000	0.6212	1.0000
Cascade–Borealis	0.5576	0.9818	1.0000
Cascade–Space	1.0000	1.0000	1.0000
Borealis–Space	0.2701	0.8923	0.5333

**Table 3:** *p*-values of the Wilcoxon signed-rank tests performed to compare the SPSL answers during the different phases of the experiment. T1 and T8 represent the surveys at the end of the resting state periods. T2, T3, and T4 correspond to the three surveys taken at three different instants during the MIST phase. T5, T6, and T7 refer to the three surveys that we conducted in the relaxation phase. The asterisks (*) indicate significant differences between the answers given in two surveys.

Instants	T1-T2	T2-T3	T3-T4	T4-T5	T5-T6	T6-T7	T7-T8
*p*-value	0.0003 *	0.0020 *	0.2891	0.0001 *	0.0078 *	1.0000	0.4531

## Data Availability

The data presented in this study are available on request from the corresponding author. The data are not publicly available due to preserve the privacy of the participants.
